# Omega-3 fatty acids decreased irritability of patients with bipolar disorder in an add-on, open label study

**DOI:** 10.1186/1475-2891-4-6

**Published:** 2005-02-09

**Authors:** Kemal Sagduyu, Mehmet E Dokucu, Bruce A Eddy, Gerald Craigen, Claudia F Baldassano, Ayşegül Yıldız

**Affiliations:** 1 University of Missouri – Kansas City, Missouri, 8801 West 148^th ^Terrace, Overland Park, KS 66221, USA; 2 Washington University, School of Medicine, Department of Psychiatry, Campus Box: 8134, 660 South Euclid Avenue, St. Louis, Missouri, 63110, USA; 3 Department of Psychiatry, School of Medicine, University of Missouri-Kansas City, Resource Development Institute, 601 Walnut Street, Kansas City, MO 64106, USA; 4 Mood Disorders Psychopharmacology Unit, University Health Network, Toronto Western Hospital, 399 Bathurst Street, ECW-3D-010, Toronto, Ontario M5T 2S8, Canada; 5 Mood and Anxiety Disorders Clinic, Department of Psychiatry, University of Pennsylvania, 3535 Market Street, 2nd floor, Philadelphia, PA 19104, USA; 6 Dokuz Eylül Medical School, Department of Psychiatry, İzmir, Turkey

## Abstract

This is a report on a 37-patient continuation study of the open ended, Omega-3 Fatty Acid (O-3FA) add-on study. Subjects consisted of the original 19 patients, along with 18 new patients recruited and followed in the same fashion as the first nineteen. Subjects carried a DSM-IV-TR diagnosis of Bipolar Disorder and were visiting a Mood Disorder Clinic regularly through the length of the study. At each visit, patients' clinical status was monitored using the Clinical Monitoring Form. Subjects reported on the frequency and severity of irritability experienced during the preceding ten days; frequency was measured by way of percentage of days in which subjects experienced irritability, while severity of that irritability was rated on a Likert scale of 1 – 4 (if present). The irritability component of Young Mania Rating Scale (YMRS) was also recorded quarterly on 13 of the 39 patients consistently. Patients had persistent irritability despite their ongoing pharmacologic and psychotherapy.

Omega-3 Fatty Acid intake helped with the irritability component of patients suffering from bipolar disorder with a significant presenting sign of irritability. Low dose (1 to 2 grams per day), add-on O-3FA may also help with the irritability component of different clinical conditions, such as schizophrenia, borderline personality disorder and other psychiatric conditions with a common presenting sign of irritability.

## Introduction

According to the United States National Institute of Mental Health (NIMH), Bipolar Disorder (BPD), also known as manic-depressive illness, is a serious medical illness that causes shifts in a person's mood, energy, and ability to function. Different from the normal ups and downs that everyone goes through, the symptoms of bipolar disorder are severe. Bipolar disorder is a complex, chronic condition associated with considerable morbidity and mortality, including a high rate of suicide. Bipolar disorder causes dramatic mood swings from overly "high" and/or irritable to sad and hopeless, and then back again, often with periods of normal mood in between. Severe changes in energy and behavior go along with these changes in mood. The periods of highs and lows are called episodes of mania and depression. Most people with bipolar disorder can achieve substantial stabilization of their mood swings and related symptoms over time with proper treatment. A strategy that combines medication and psychosocial treatment is optimal for managing the disorder over time.

## Background

Omega-3 fatty acids (0-3FA) may have a beneficial effect on irritable mood. Low O-3FA levels in red blood cell membranes of depressed patients hint that O-3FA may be helpful in treating mood disorders [1]. A recent article has given an excellent review of O-3FA and studies showing their effectiveness in depression, bipolar disorder and aggression [2]. In this article, two published studies are discussed that have reported on similar therapeutic effects of O-3FA [2]. One placebo-controlled study of 20 patients revealed that ethyl ester of eicosapentaenoic acid (E-EPA) was effective in stabilizing the moods of depressed patients [3]. Another report, a double-blind, placebo controlled study (N = 22/19), measured the effect of O-3FA docosahexaenoic acid (DHA) on the aggressive tendencies of college students. The O-3FA DHA group (1.5–1.8 g O-FA DHA/day) did not display any increase in aggressive tendencies when external stressors peaked, while the placebo group displayed a significant increase in their aggressive tendencies under similar circumstances [4]. In a recent study, 25% of 111 patients with bipolar-I disorder who met criteria for a DSM-IV major depressive episode also experienced substantial irritability in the absence of associated symptoms of mania. These findings suggest that abnormal irritability is not limited to mania or mixed states [5]. However, recent studies give caution that at a 6 gram per day average daily dose, as a single agent, omega 3 fatty acids may not be as effective as an antidepressant [6-9].

O-3FA may also help with the irritability component of different clinical conditions, such as depression, mania, schizophrenia, borderline personality disorder and other psychiatric conditions with a common presenting sign of irritability. Numerous other conditions have an irritability component, including Borderline Personality Disorder, Alzheimer's disease, Premenstrual Dysphoric Disorder, to name a few [10-12]. There is one report suggesting beneficial effect of Omega-3 Fatty acid treatment for Borderline Personality Disorder. This double-blind, placebo-controlled pilot study specifically showed that EPA may influence both aggression and depression [12]. Although attention-deficit/hyperactivity disorder (ADHD) also has an irritability component, recent publications bring doubt to the O3FA connection in ADHD [13,14].

A recent, open ended, O-3FA add-on study has shown beneficial effect of O-3FA on irritability in 19 patients with mood disorders [15]. These patients had already been receiving different combinations of pharmacotherapy and talk therapy. Despite their treatment, the irritability component of their illness was still causing social, occupational and other life disturbances. Hence, they were chosen for the O-3FA add-on component of the study. In the nineteen-patient phase of the study, bipolar patients of every subtype, ages 18 to 65 years, with significant irritability were studied. All patients received a systematic assessment battery at entry and were treated by a psychiatrist, trained to deliver care and measure outcomes in patients with bipolar disorder, consistent with expert recommendations. At every follow-up visit, the treating psychiatrist completed a standardized assessment and assigns a clinical status based on DSM-IV criteria. Patients had independent evaluations at regular intervals throughout the study and remain under the care of the same treating psychiatrist while receiving variable medications and talk therapy, depending on their need [15]. In the 19-patient study, a paired sample t-test revealed a large decrease in the percent of days irritable after O-3FA was administered. Before treatment, the mean irritability percentage was 81.05 (SD = 23.31) and after treatment the mean irritability percentage dropped to 30.00 (SD = 36.67). Despite the small number of patients in the study (n = 19), the difference between means was statistically significant (t (18) 4.512, p < .001). Using a paired sample t-test, a significant difference was also found between the highest irritability score (mean = 2.79; SD = 0.92) and the last recorded irritability (mean = 0.79; SD = 0.85) while taking O-3FA (t(18) = 8.270; p < .001) [15].

## Methods

This is a report on a 37-patient continuation phase of the open ended, O-3FA add-on study. Subjects consisted of the original 19 patients, in addition to the 18 new patients recruited and followed in the same fashion as the first nineteen [15]. Subjects carried a DSM-IV-TR [16] diagnosis of Bipolar Disorder and were visiting a Mood Disorder Clinic regularly throughout the length of the study. At each visit, patients' clinical status was monitored using the Clinical Monitoring Form [17]. Subjects reported on the frequency and severity of irritability experienced during the preceding ten days; frequency was measured by way of percentage of days in which subjects experienced irritability, while severity of that irritability was rated on a Likert scale of 1 – 4 (if present). The irritability component of Young Mania Rating Scale [18] (YMRS) was also recorded quarterly on 13 of the 39 patients consistently. The patients were asked about general dietary omega-3 intake before the fish oil was added on, and basic nutritional guidance was given to subjects at the clinic. Patients in general were not heavy fish/product consumers.

### Dosage

Starting dose and last maintenance dose were available for 37 subjects (Table 1). Subjects self-medicated, and therefore, the last maintenance dose of O-3FA was chosen by each subject. The mean starting dose was 1824.32 mg (SD 1075.07), and the mean for the last maintenance dose was considerably higher at 2878.38 mg (SD 2011.79). The increase was statistically significant using a paired sample t-test (t = -3.44, 36df, p = .001).

## Statistical Results

### Percentage of Irritable (Days)

The initial mean was 63.51 (SD 34.17), indicating that on average, subjects were irritable for about six of the previous ten days. The mean for the last recorded percentage was less than half of the initial score: 30.27 (SD 34.03). The decrease was found to be statistically significant using a paired sample t-test (t = 4.36, 36 df, p < .001). The difference between the distributions was examined using the non-parametric sign test. The number of negative differences (25) significantly exceeded positive differences (7); there were five ties, and the pre/post distributions were significantly different (p < .003).

### YMRS Irritability Sub-score

Thirty four subjects had initial and last recorded YMRS irritability sub-scores. As with the above means there was a sizable decrease. The initial mean score was 3.18 (SD 1.09). The mean for the last recorded percentage was 1.68 (SD 1.89). The decrease was found to be statistically significant using a paired sample t-test (t = 4.21, 33 df, p < .001).

### YMRS Total Score

Starting and last recorded YMRS scores were available for 34 subjects. The mean starting score 10.71 (SD 6.77), and the mean for the last recorded score was 4.85 (SD 5.63). The decrease found to be statistically significant using a paired sample t-test (t = 4.14, 33 df, p < .001).

### Severity

Thirty six subjects had initial and last recorded severity scores on the ADE. Again, a decrease was found. The initial mean score was 2.14 (SD 1.22). The mean for the last recorded score was 0.94 (SD 0.92). This decrease was found to be statistically significant using a paired sample t-test (t = 5.23, 35 df, p < .001).

### Composite: Severity and Irritability

As an exploratory measure, a composite score was created by multiplying the ADE severity score, which has a maximum of 4 points, by the percentage of the ten days prior to measurement which the patient was rated as irritable. The initial mean on this composite was 159.72. As with other measures, there was wide variation: SD = 122.92. The mean for this measure on the last recorded scores was percentage was about one-fourth of the initial score: 43.89 (SD 64.38). The decrease was found to be statistically significant using a paired sample t-test (t = 5.00, 35 df, p < .001).

### Last Recorded Maintenance Dose and Percentage of Irritability After

Because of apparent wide variation on these two measures and a concern that outliers may have affected some results, the last recorded irritability scores were plotted against the maintenance dose. This revealed a rather bimodal pattern, in which relatively lower irritability measures (≤ 50%) clustered in the quadrant with lower dosage levels (≤ 4,000 mg).

### Duration and YMRS Total

In response to a similar observation regarding wide variation in the last recorded values (84 days to 5.5 years) the values were also plotted. A clearly bimodal pattern appeared in which 11 subjects (about one-third of study participants) clustered in the quadrant representing short duration (<500 days) and higher YMRS totals (>7). The remaining two-thirds of subjects clustered in the quadrant representing short duration and lower YMRS totals (<6).

### Subject Weight

The mean start weight was 176.97 lbs (SD 43.13), and the mean for the last weight recorded was slightly higher at 178.59 lbs (SD 43.24). The increase was not statistically significant.

## Follow-up Subjects

Follow-up information, recorded after the collection of the "last" scores for most of the above variables, was available for 13 of the 37 subjects. Final YMRS total or scale scores were not available for this sub-group.

### Omega 3 Duration

The final date recorded for the duration of O3 was derived based from an O3 start date and a "final" date recorded for O3. The time period ranged 84 days to 1995 days (5.46 years). The mean duration of O3 for this group was 439.62 days (SD = 487.46).

### Dosage

For these subjects, the mean starting dose was 1807.69 mg (SD 990.34), and the mean for the last maintenance dose was higher at 2615.38 mg (SD 1894.66). The increase was not significant.

### Percentage Irritable (Days)

The initial mean was 82.31 (SD 20.88). The mean for the last recorded percentage was dramatically lower: 25.38 (SD 32.04). The decrease was found to be statistically significant using a paired sample t-test (t = 6.52 12 df, p < .001). The difference between the distributions was examined using a sign test. The number of negative differences (12) significantly exceeded positive differences (0); there was one tie, and the pre/post distributions were significantly different (p < .001).

### Severity

The initial mean score for the 13 subjects with final scores was 2.69 (SD 0.95). The mean for the final score was 0.77 (SD 0.83). This decrease was found to be statistically significant using a paired sample t-test (t = 6.22, 12 df, p < .001).

### Composite: Severity and Irritability

An exploratory composite score, described above, was also created for the subjects with final scores. For these subjects, the initial mean was higher than that of the total group, 223.08. Again, there was wide variation: SD = 104.19. The mean for this measure on the last recorded scores was percentage was much lower that the initial score: 33.08 (SD 39.87). The decrease was found to be statistically significant using a paired sample t-test (t = 6.70, 12 df, p < .001).

### Weight

For these 13 subjects, the mean start weight was 166.23 lbs (SD 35.68), and the mean for the final weight recorded was also slightly higher at 168.23 lbs (33.62). As with the previous finding regarding weight, the increase was not statistically significant.

## Results

Omega-3 Fatty Acids added onto the existing treatment helped with the irritability component of a significant percentage of patients suffering from bipolar disorder with a persistent sign of irritability.

## Discussion

As seen from the standard deviations of several of the variables discussed here, measures ranged widely. This creates difficulty in using descriptive data, such as means, to adequately portray subject attributes and performance. Using data reduction techniques or grouping subjects according to high and low scores on various attributes may be one way to increase the descriptiveness, which would be possible and more reasonable with a larger pool of subjects.

A potential limitation or interpretive consideration merits discussion. For many of the variables discussed above, noticeable differences in measures were observed between the "starting" versus "last recorded" group (n = 37) and the "starting" versus "final" measures group (n = 13). Given these differences and the smaller number of subjects in the second set of comparisons, "starting" versus "final" comparisons should be interpreted with caution until differences inherent in this "final" subgroup (n = 13) are more clearly understood. This is clearly seen in the results of sign tests, in which the apparent magnitude of the "final" effects is pronounced.

Statistically significant within-subjects differences were found in several independent variables. This is especially notable given the small number of subjects. The preliminary findings suggest that a rigorously designed study tailored especially to the examination of the effects of O-3FA is warranted.

The majority of data were collected within an ongoing "best-practice, outpatient bipolar disorder study" that involved medications and talk therapy which we have not reported or discussed herein. Results must, therefore, be interpreted with caution.

There are several mechanisms through which O-3FA are theorized to help with mood, irritability, aggression etc. Suggested theories of mechanism converge on the theory of nerve cell membrane stabilization. A recent study has come closest to showing physical proof of effectiveness of O-3FA through indirect demonstration of greater membrane fluidity, as detected by reductions in Tesla-2 (T2) values in MRI scans [19]. The overlapping beneficial effects of antipsychotics, antidepressants, anticonvulsants, O-3FA, and nonpsychoactive cannabinoids, as they relate to pain, stroke, schizophrenia, psychoneuroimmunology, Alzheimer's disease, and stress, may be because of their common effects at protein kinases, thus affecting the structure and function of the cell membrane and the cell [20]. These changes should help the cell operate within an optimal level of excitation, which may be related to emerging evidence that these therapeutic agents have neuroprotective value [20]. A recent randomized placebo controlled double blind intervention study suggests an adaptogenic role for O-3FA in stress [21].

We would like to discuss briefly the issue of daily dosing of O-3FA for nutrition and medicinal purpose: Recent studies give caution that at a 6 gram per day average daily dose, as a single agent, omega 3 fatty acids may not be as effective as an antidepressant [6-9]. However, these studies may have given too high of a dose of O-3FA, above 6 grams daily, with possibly beyond a therapeutic window of effectiveness for O-3FA. Our scatter plots indicate that the optimum effective dose for irritability is at 1–2 gram of EPA plus DHA per day, which would be the dosing we suggest. A recent exploratory dose study of O-3FA for schizophrenic patients showed that 2 g/day EPA-treated patients had lower symptom scores, and needed less medication greatest. In this study, there was a positive relationship between improvement on rating scales and rise in red blood cell arachidonic acid concentration as well [22].

The United States (US) accounts for more than 51% of the 430.3 billion dollar expended on pharmaceutical products worldwide each year [23]. World healthcare society first needs access to low-cost, nontoxic, non-expert-dependent interventions to ensure basic health outcomes. Food may represent the most cost-effective means of promoting public health [23]. The American Heart Association recommends consumption of two servings of fish per week for persons with no history of coronary heart disease and at least one serving of fish daily for those with known coronary heart disease [24]. Approximately 1 g per day of EPA acid plus DHA acid is recommended for cardioprotection [24]. Higher dosages of omega-3 fatty acids are required to reduce elevated triglyceride levels (2 to 4 g per day) and to reduce morning stiffness and the number of tender joints in patients with rheumatoid arthritis (at least 3 g per day) [24].

We conclude that it is beneficial in many ways to establish a regular intake of 1–2 g per day EPA acid plus DHA, similar to daily intake of vitamins with minerals. Dietary interventions to remedy omega-3 deficiency is necessary [23]. It is time for more aggressive funding for research into medicinal foods, such as omega-three fatty acids [23].

## Figures and Tables

**Table 1:** Initial, Last and Final Omega 3 Dosages (mg). summarizes dosage under three conditions. Figures for the Initial Dose include two subjects (n = 39) for whom no corresponding follow-up data were available.

Initial, Last Recorded and Final Omega 3 Dosages (mg)			
	Initial	Last Recorded	Final^T1^

n	39	37	13
Mean	1833.33	2878.38	2615.38
Mode	1000^T2, T3^	1000	2000
Median	2000	2000	2000
SD	1071.91	2011.79	1894.66

T1 = Final group results (n = 13) are discussed below.

T2 = Multiple modes exist. The smallest value is shown.

T3 = One gram (1,000 miligram) of fish oil; of which about 180 milligrams is (eicosapentaenoic acid) EPA and 120 milligrams is DHA (docosahexaenoic acid), (for a total of 300 milligrams of omega 3's) in each clear capsule.
